# "The Hospital" Nursing Mirror

**Published:** 1901-03-02

**Authors:** 


"The Hospital" Nursing Mirror.

Being the Nursing Section of "The Hospital."

[Contributions for this Section of "The Hospital" should be addressed to the Editor, "The Hospital" Nursing Mirror, 28 & 29 Southampton Street Strand, London, W.C.]

### Notes on News from the Nursing World.

### QUEEN VICTORIA'S JUBILEE INSTITUTE FOR NURSES.

The Duke of Portland and other officials of Queen Victoria's Jubilee Institute for Nurses have addressed a letter to the committee, who have under consideration the question of the National Memorial to Queen Victoria, asking that the national charity with which they are connected may form some part of it. They base their claims for consideration on the grounds that the charity was founded by Queen Victoria herself; that it reaches the very poorest in England, Ireland, Scotland, and Wales, and is therefore national; that it is absolutely undenominational, and includes nurses of all religions, who minister to the sick poor without distinction of creed; that all the machinery is to hand for affiliating similar schemes in the Colonies, and that in this way the National Memorial could be enlarged to an Imperial one.

### A REPROACH TO BIRMINGHAM.

Why is such an enterprising city as Birmingham so slack in supporting its admirable District Nursing Society, which has reached the twenty-ninth year of its existence, and under whose auspices 58,000 visits were paid in 1900? The increase in the work of the organisation since 1894 has been extraordinary; but though the work has more than doubled, the number of nurses has not increased in the same proportion, and the income was less last year than it was in 1899. It is a reflection on the liberality of the manufacturers of Birmingham that the public subscriptions only amount to £579. Contrasted with Manchester, which raises upwards of £3,000 for a similar purpose, this is very small; and the plea that there are so many other claims cannot be accepted as valid. The Lord Mayor of Birmingham is satisfied that manufacturers in that city recognise the benefit of health to their workpeople, "because very many of them give a good deal of attention to the sanitary condition of their establishments." But why not also give a reasonable sum of money to the society which confessedly ministers successfully to the needs of the workmen when they are sick? It is not stated in the report whether the men contribute to its maintenance, but masters and men together must be able, with the slightest self-denial, to find more than the paltry total of £579.

### A MATRON ON THE NURSING VOCATION.

A passage in a letter from "A Matron of Experience" in another column cannot be passed over in silence. But for the authorship of the communication, the assertion of the writer that "there is a tendency in the present day among men of the world, and the clergy too, to rank a nurse either as a nun or a demi-mondaine" would hardly, perhaps, merit notice. That the matron of a hospital should make it is rather startling. The charitable conclusion is that, having come across some very objectionable men of the world and some very queer specimens of the clergy, she thinks that they are representative of their class. We assure her that this is not the case. The average man of the world has a genuine respect for the nurse who unostentatiously does her duty, and the average clergyman welcomes her as a co-worker in the best of all causes. But we fear that a matron who allows herself to write of a nun as an "unsexed nonentity," and brands a man who suggested that a nurse might possibly dine with the housekeeper in a large hotel as not a safe patient for her to have anything to do with, is hopelessly blinded by prejudice.

### RESIGNATION OF A LADY SUPERINTENDENT AND NURSING STAFF.

Miss Baxter, the lady superintendent of the County and City of Cork Hospital for Women and Children, resigned her appointment last week, and on the evening of the same day, without any consultation, the nursing staff also intimated their intention to resign. The reason for these resignations, which will take effect in three months, is certain criticisms passed upon the management of the hospital at a meeting at which it was decided to endow the institution as a memorial of Queen Victoria. These criticisms are regarded by the lady superintendent as a reflection on herself, and the nursing staff entirely share her opinion. But in view of the fact that Miss Baxter's great services have repeatedly been acknowledged by the committee of management, and of her popularity both in the hospital and in the town, we cannot help thinking that a *modus vivendi* may yet be found. It would be a bad start for a memorial of Queen Victoria to be associated, at the time of its origin, with a quarrel which might, as far as we can judge, be easily adjusted by the exercise of a little tact and good sense.

### NEWTON ABBOT INFIRMARY.

A temperate letter from Miss Flaherty, the nurse at Newton Abbot Infirmary who has been so strangely treated by the Guardians, appears in another column. The letter speaks for itself, but we may add that from other sources we have received communications expressing warm sympathy with the writer. In fact, it may be said that the feeling in the town is on her side, and that the Guardians are very much blamed for the action they have taken in the matter. A constant visitor to the Infirmary speaks of Miss Flaherty as a very good and skilful nurse, "who has done nothing to deserve the mud flung at her by common gossip." Happily, it will not stick.

### NURSES AND CHAMPAGNE.

What is known in the county of Durham as "the Chester-le-Street Hospital Scandal" has ended in the compulsory resignation of the nurses who were apparently hopelessly at warfare with each other. It was obvious that they could not both remain; and a careful perusal of the statements at the meetings of the Rural District Council leads us to the conclusion that the allegations of the nurse-matron against her subordinate were not sustained, and should not have been made. The admitted payment, by the former, of five shillings to a laundrymaid, which she says was for "extra washing," but which the laundrymaid herself avers was given to her to watch Dr. Taylor and Nurse Manson, is an incident forbearingly described by the Chairman of the Council as "unusual." Perhaps, in the circumstances, it was as well that Nurse Manson should also leave, though her character remains unaffected by the "scandal." We trust that the lesson of the case will not be lost upon Dr. Taylor, who was falsely accused of drinking champagne and causing it to be drunk at the expense of the ratepayers. But it is true that on three special festive occasions he treated the staff to a small quantity; and on this point we think that, if it is no offence for a nurse to take a glass of champagne, it is undoubtedly an indiscretion for the doctor at the hospital in which she is at work to introduce wine for purposes of conviviality.

### PORTSMOUTH WORKHOUSE INFIRMARY.

As the result of the inquiry of the Infirmary Committee of the Portsmouth Board of Guardians with respect to the unpleasantness which resulted in the retirement of the matron and assistant matron, a report has been presented. The committee find that the causes of discontent "are not of a serious nature." They are thus enumerated:—"That the nurses do not get their whole day off once a month, as under the present arrangements they have to do duty in the ward until 10 A.M.; that the charge nurses object to dining at the same table as the probationers; that if out on late leave they get no supper upon their return to the institution; that the eggs and bacon were both occasionally unsatisfactory; that the sitting-room should more often contain two fires; that the bedrooms are too cold, the thermometer registering 40 degrees; that they should be allowed coffee for supper." The committee, it was stated at the meeting of Guardians by whom the report was discussed, did not acknowledge that all the complaints were reasonable, but expressed the opinion that "if these grievances exist they should, so far as practicable, be remedied." Nothing could be more cautious than this opinion, and Mrs. Proctor very properly said that if these grievances existed they should be dealt with by the committee, and should not have been allowed to result in a disturbance. She added her conviction that there is "still something behind," which is, of course, very probable. Our view of the enumerated causes of discontent is that they should receive attention. "Unsatisfactory eggs and bacon" are not appetising, and a demand for more warmth, in the shape of extra fires and coffee for supper, is quite legitimate.

### THE CASE OF MISS ROGERS.

The result of the inquiry by the Local Government Board into the dispute between the East Preston Guardians and the superintendent nurse is that Miss Rogers has been directed by the Board to tender her resignation. The substance of the evidence has already been published in our columns, and it will be admitted by those who perused it that no efforts were spared to ascertain the whole of the facts.

### KINGSTON-ON-THAMES WORKHOUSE INFIRMARY.

There are now eight certificated nurses at Kingston-on-Thames Workhouse Infirmary who act as ward sisters, and eighteen probationers who receive lectures from the doctor and the superintendent nurse every week. We understand that arrangements are being made by the Guardians to separate the infirmary administration from that of the workhouse, and that the Local Government Board have sanctioned the training of probationers. A new infirmary is in course of erection, and as soon as the administrative block is completed a doctor will reside in the institution.

### NORTH STAFFORD INFIRMARY.

The fact that after an interval of only two years it has become necessary to appoint another lady superintendent of the North Stafford Infirmary raises the question whether the rules on which we commented at the time of the resignation of Miss Warburton are still in operation. It may be recollected by some of our readers that we then drew attention to the practical impossibility of avoiding friction under rules which delegated to the medical superintendent authority properly belonging to the lady superintendent; and the retirement of her successor so soon points to the conclusion that the new holder of the office will have to face the old difficulties.

### A PROSPEROUS INSTITUTE.

A word of commendation is due to the author of a suggestion made at the annual meeting of the Hampshire Nurses' Institute. As the Institute is now self-supporting it was proposed that donations should be transferred to the Jubilee Nurses' Institution, which renders valuable help to the sick poor, and is in need of pecuniary assistance. No action seems to have been taken; but we hope it is not rash to assume that the proposal, to which no opposition was offered, will be carried out. An institute which is, happily, in a position to grant a bonus of £5 5s. to the matron, £1 1s. each to the eight senior staff nurses, and 10s. 6d. to each of the juniors, may fitly dispense with subscriptions.

### PEACE AT MALDON.

The wreckage of the Maldon Nursing Association has been avoided, and things have been put on a fresh basis. We are glad to see that an attempt to make the single nurse the Association is at present able to afford responsible for maternity as well as for general cases, was not successful. As it was pointed out at the election of new officers and of the committee, there are strong objections, from a medical point of view, to a maternity nurse attending general cases. It was also rightly held that the duty of the Association is, first of all, to take care that poor people who require a nurse receive proper attention before a nurse is provided for maternity cases, which are paid for. Now that peace has been restored at Maldon, it should not be long before the funds of the Association are sufficient to warrant the engagement of a second nurse for maternity cases. The fees would partly supply her salary.

### A NURSE WHO MARRIED BENEATH HER.

At the Derby Assizes on Monday Mary Ann Coulan, described as a hospital nurse, was charged with contracting a bigamous marriage with James Wilkinson at Buxton on March 21st, 1899, her husband, to whom she was married at Manchester on September, 1893, being alive. The evidence showed that the prisoner and her first husband were natives of the same village in County Monaghan. She occupied a much better social position, and at first refused his offer of marriage. Afterwards she went to Manchester. Conlan followed, and succeeded in persuading her to marry him. It was a marriage in name only, because Conlan went straight back to Ireland, and the parties did not live together as man and wife. In 1897 Conlan again came to England. This time the couple lived together for one week only at Southport, where the prisoner paid all the expenses. Then they quarrelled over religious matters, and henceforth held no communication with each other. Subsequently prisoner was employed at a Buxton hospital, where she met Wilkinson, who also came from her native village in Ireland. Prisoner never revealed the fact that she had been married, and went through a form of marriage with Wilkinson. They visited Ireland last year, and Conlan then gave information to the police. The prisoner's counsel suggested that the man Conlan set the prosecution on foot because his allowance had been stopped, and Mr. Justice Lawrence, taking a very lenient view of the offence, sentenced the prisoner to one day's imprisonment.

### HOUSE-TO-HOUSE COLLECTION.

The annual meeting of the Willington-on-Tyne Nursing Association has just been held. It was started on February 1st, 1900, and maintains two nurses who attended 150 cases last year, and paid nearly 4,000 visits. The house-to-house collections obtained £100 17s., and the Association has in hand this month the handsome balance of £49. The substantial results obtained at Willington-on-Tyne should encourage other organisations to enlist the services of house-to-house collectors.

### THE COMING CONGRESS OF NURSES AT BUFFALO, U.S.A.

The committee who are responsible for convening a congress of nurses at Buffalo, U.S.A., in September have sent letters asking for delegates to the following organisations in Great Britain and Ireland:—The Royal Navy Nursing Service, the Army Nursing Service, the India Nursing Service, the Poor Law Nursing Service, the Metropolitan Asylums Board, the Queen's Institute for Nurses (both the central office and the branches in Ireland, Scotland, and Wales), the Colonial Nursing Association, the Northern Workhouse Association, the Matrons' Council of Great Britain and Ireland, the League of St. Bartholomew's Nurses, the Royal British Nurses' Association, the Registered Nurses' Society, St. John's House, the Nurses' Co-operation, the Midwives' Institute, the Incorporated Society of Trained Masseuses, the Bradford Incorporated Nurses' Institution, the Dublin Nurses' Club, the Dublin Metropolitan Technical School for Nurses; and to the London, St. Bartholomew's, the Middlesex, St. George's and St. Thomas's hospitals in London; the Birmingham General Hospital, the Leeds General Infirmary, the Royal Infirmaries of Edinburgh and Glasgow, the Western Infirmary of Glasgow, and Sir Patrick Dun's Hospital, Dublin. It has been decided that Miss Florence Nightingale should be asked to accept an honorary title, and that representative names of the different countries should be placed on the list of honorary officers.

### IN AID OF A NURSING INSTITUTION.

A bazaar in connection with the Haggerston and Hoxton District Nursing Association is to be held at the Shoreditch Town Hall on May 8th, 9th, and 10th, in order to clear off the remaining debt on the necessary enlargement of the Nurses' Home. As the district in which the nurses work is one of the poorest, outside help is greatly needed, and any contributions of work or money will be most gratefully received by Miss Böege, Nurses' Home, 80 Nichols Square, Hackney Road, N.E.

### BARBADOS GENERAL HOSPITAL.

According to a cutting which reaches us from Barbados, the acting secretary reported at the quarterly directors' meeting on November 14th the receipt of "a copy of 'The Hospital' Nursing Mirror in which the directors' reply to the article which appeared in that journal in its issue of July 21st was published." From the same cutting it seems that Miss Agnes Veecock is the latest and only applicant for the appointment of head nurse, but it is not stated whether she has received it.

### BOYCOTTING AN IRISH NURSE.

A nurse in an Irish town has been boycotted by the people through fear of sickness, and has consequently refused to continue in attendance on the ambulance van. The guardians talk of compelling her to fulfil her contract, but they may not find it an easy matter to succeed in their task. The nurse should do her duty under any circumstances, but it is very silly of the townspeople to boycott her. If she takes proper precautions, there is no reason why she should be a centre of infection.

### NORTHAMPTON NURSES.

At the annual meeting of the Northampton Town and County Nursing Institution, which embraces a district fund and a private nursing department, the reports were adopted. The accounts are kept quite separate, but the dual system is advantageous to the district fund, which is associated with Queen Victoria's Jubilee Institute, because it only bears a third of the total establishment expenses. Last year the visits paid by the district nurses were upwards of 11,500, and a pleasing feature of the work is that the greater portion of the calls came from the medical men in the town. Dr. Brand in supporting the motion for the adoption of the report said, "that some of his very poor patients told him that the nurses must be angels in disguise. And he quite agreed with them." A feature of the report of the parish nursing department is that during the twelve months fourteen cases were attended at reduced charges to people who were unable to pay full fees. Both organisations will gain substantially by the opening of the new institute, which, thanks to the Diamond Jubilee Committee, is to be their joint home.

### CITY OF LONDON LYING-IN HOSPITAL.

The new dormitories and sitting-rooms for nurses which were ready for occupation at the beginning of the year have been found of the greatest service. The total cost of the building and furnishing amounted to £3,460, and it is satisfactory to learn from the report that the whole of this amount has been paid without drawing heavily on the capital account.

### SHORT ITEMS.

The appointment of Miss Neech as matron of the Hertford Hospital at Paris was announced some time ago in our columns. She has under her six nurses, all of whom have been engaged from England.—An appeal is being made by the military governor of St. Michael's Sisterhood, Bloemfontein, for aid for that institution, which is now in great financial straits, owing partly to the fact that during the war many enteric patients were nursed by the sisters in the building, and parents cannot, or will not, send their children as before. The Rev. E. Elwes, Woolbeding Rectory, Midhurst, is acting as hon. treasurer for the Sisterhood in England.—On Thursday, March 7th, a matinée in aid of the National Orthopædic Hospital, Great Portland Street, will be given at the Lyceum Theatre A very attractive programme has been arranged.—At a meeting of the Dorset Home for Nurses at Dorchester this week Mr. Hamilton Palairet, chairman of the committee, stated that subscribers and friends had collected £118 for the funds of the Imperial Yeomanry Hospital.—Miss A. J. Downing, of Forest View, Enfield, who died on the 20th of last January, has left a bequest to the Mildmay Nursing Institute.

### Lectures to Nurses on Medicine and Medical Nursing.

By NORMAN DALTON, M.D. London, F.R.C.P., Physician to King's College Hospital.

(*Continued from page* 260.)

LECTURE V. FEVER—(*continued*).

THE TYPHOID STATE—THE NURSING OF THE FEBRILE CONDITION.

*The Typhoid State.*—This diseased condition is closely associated with the pyrexial state, although not an essential part of it. It consists of a group of symptoms which usually develop and reach their full extent in three or four days, but sometimes much more rapidly. The earliest signs of it are that the patient becomes restless at night, talks in his sleep, and probably dreams a great deal. He may try to get out of bed, under the impression that he has an important engagement to keep. Later on his mind is confused even when he is awake; and then he becomes delirious, muttering incoherently to himself. For a time it may be possible to rouse him; for instance, he can often be got to put out his tongue when spoken to sharply. Finally he becomes completely unconscious. In the meantime his tongue has been becoming drier and drier, and brown streaks appear on it, and it is tremulous when protruded. Eventually it may become quite brown or black, like the tongue of a parrot. The black crusts on the tongue are called sordes, and similar black crusts may form on the lips. From the beginning the patient may be restless, but as the unconsciousness deepens he begins to pick at the bedclothes or at imaginary objects in the air. The motions and urine are passed into the bed, but in some cases the urine is retained, when the patient first becomes unconscious, and the catheter may have to be used more than once.

It will be noticed that the symptoms of the typhoid state develop gradually, so that on seeing a patient on one day, and finding his tongue rather dry, and learning that he was rather "wandering" during the night, we conclude that the typhoid state is coming on. On the next day it may have developed more, or it may have passed off, according as the disease in which it is occurring has got worse or better. When it has become fully developed the patients frequently die; but if they recover the symptoms pass off gradually.

The typhoid state is most frequently met with in the specific fevers, and especially in those fevers, such as typhus and typhoid, in which the temperature remains high for a long time. It may be met with in small-pox, when the secondary fever is prolonged, in general tuberculosis, and in pyæmia. It is not often seen in scarlet fever, diphtheria, or pneumonia, diseases which do not last very long; but still, if the amount of poison in the blood in these cases be very great, or if, through complications, the fever remains high for a long time, it may develop. In fact, it may be said that, in any disease whatsoever, if the temperature remain over 103° without remissions for four or five days, symptoms of the typhoid state are almost sure to set in. On the other hand, we know that it does not depend simply on the heat of the body, for it is very conspicuous in a disease called acute yellow atrophy of the liver, in which the temperature may even be subnormal.

The "nursing" of the febrile state to a great extent depends on the particular disease in which it occurs, and will be treated of in the lectures on the specific fevers. But some points may be mentioned now. In taking the temperature nurses should be careful to shake the mercury down to 97° before introducing the thermometer, because it is quite as important to note subnormal temperatures as high ones. As regards the average normal temperature in different parts of the body, it is said to be 98·3 in the mouth, 98·4 in the axilla, and 98·9 in the rectum.

The points to be observed about the pulse are its strength and the number of beats per minute. When a four or six hourly chart is being kept, the pulse should always be counted when the temperature is taken, and the rate put down on the chart. The reason for this is that, whereas in most fevers, such as typhoid, the temperature and pulse are fairly proportional to each other (a temperature of 100° going with a pulse of about 85, and so on), in the fever of acute general tuberculosis and of ulcerative endocarditis, which are very difficult diseases to diagnose, the proportion may not be kept; for instance, the pulse may be very rapid, with quite an average temperature.

The dieting of fever patients is usually strictly regulated by the physician. As a general rule fever patients should be waked to take their feeds and brandy at the regular time. The quantity of food taken in the twenty-four hours should always be noted.

Nurses should always be on the look-out for complications. While washing the patient they have an opportunity of observing the skin, and should look out keenly for any rashes, or spots, or sore places, and report their presence on the next visit. Bed-sores can often be prevented by employing a water-bed from the first, by toughening the skin with spirit lotion, and by keeping the buttocks scrupulously clean. Should any redness appear on the sacrum, the breaking of the skin may be combated by the adjustment of water-pillows, the skilful arrangement of which can only be learnt at the bedside. The occurrence of a cough is important, as it may be the first sign of a lung complication, and pain in any part of the body should always be noted. In all prolonged fevers nurses should be on the alert for any symptoms of the typhoid state because the earliest signs of that condition usually appear at night, when the nurse is alone; and although they may have passed off by the time the doctor pays his visit in the morning, it is important for him to know that they have occurred. So that any talking in the sleep, or incoherence when awake, or any dryness of the tongue must be carefully noted. When the patient is unconscious in the typhoid state it must be remembered that although the urine may be dribbling into the bed, the bladder may be quite full. Other points will be mentioned in the lecture on the fevers. Nurses should also be on the look-out for favourable signs; for instance, patients in the typhoid state most often lie on their back, and should they turn on their side and assume a more comfortable attitude the sign is a good one.

### "The Hospital" Convalescent Fund.

THE Hon. Secretary begs to acknowledge with grateful thanks the receipt of £1 from Mr. Erskine.

### To Nurses.

**WE invite contributions from any of our readers, and shall be glad to pay for "Notes on News from the Nursing World," or for articles describing nursing experiences, or dealing with any nursing question from an original point of view. The minimum payment for contributions is 5s., but we welcome interesting contributions of a column, or a page, in length. It may be added that notices of entertainments, presentations, and deaths are not paid for, but, of course, we are always glad to receive them. All rejected manuscripts are returned in due course, and all payments for manuscripts used are made as early as possible at the beginning of each quarter.**

### Nursing Miners in Johannesburg and Soldiers at Sea.

BY A CORRESPONDENT.

SISTER CLAPHAM, who has just arrived in England for the third time during the war in South Africa, describes herself as a "Colonial Volunteer." She is, however, English-born, and comes from Yorkshire.

### AT THE MINES HOSPITAL.

"I have been nursing in the Mines Hospital," she told me in the course of a chat, "at Johannesburg. Most of the mines have hospitals on account of the many accidents, and at Johannesburg we have patients from six mines. We get all kinds of men, but the Englishmen are mostly from Cornwall."

"Is it a large hospital?" I asked.

"There are three wards, one of which is for Kaffirs."

"What sort of patients are they?"

"Very good; they make no fuss."

"The number fluctuates, of course?"

"Yes; but we are very seldom without any at all, though there may be only two or three sometimes. From June till March is the busy time, and when the work is extra heavy we have an extra nurse from the town to help temporarily. The winter is a healthy time, and there is very little illness. There are two permanent nurses."

"The work is chiefly surgical, is it not?"

"There is a great deal of surgical work."

### VOLUNTEER WORK.

"The Mines Hospital was closed at the beginning of the war," Miss Clapham continued, "and I left Johannesburg about September 22nd, 1899, as it was nearly time for my holiday. Afterwards, being very tired of doing nothing, I went to Major McCormick and volunteered for Army work. He gave me a post as Sister on the *Lismore Castle* hospital ship, of which Sister Daly was superintendent."

I found that Miss Schwekkard, with whom an interview was published in "THE HOSPITAL" NURSING MIRROR for June 2nd, was a friend of Miss Clapham's; with Sisters Daly, Jael and Hodgebera, they had come to England in charge of one of the most seriously sick detachments of soldiers from South Africa last May—many of them just released from Ladysmith.

"The *Lismore Castle* was disbanded in August," said Miss Clapham, "and we went back to Durban, still under Government, and were sent first to Estcourt, and later to Pine Town."

"I see from the daily papers that you brought some very bad cases home in the *Manilla*."

### SOME OF THE WORST CASES.

"Yes, there were a good many," was the reply. "There was one particularly bad. An officer was shot in the spine, and paralysed from the fifth rib downwards. There were several other officers among the invalids. I had ten very sick on my side, and the Sister opposite had more."

"You shared the duty equally with Sisters Miller and Couper?"

"Yes; there were two wards, and it was remarkable how conveniently things were arranged, considering how quickly the ship had to be fitted up, for she is a transport. One ward was for cot cases and the other for convalescents. There were about seven really serious enteric cases; and I am sorry to say we lost one man, but that was from a bad heart. He became much worse after crossing the line, and died the day before we reached Plymouth. It was extremely sad."

"Did many cases develop on board?"

"Three or four men were taken very much worse after leaving Las Palmas; and one officer became sick after leaving Cape Town. It was terribly hot there when we arrived."

### A CLEVER RESCUE.

"There were fourteen lunatics on board," Miss Clapham said; "they had lost their reason through enteric and all that they had gone through. We used to go down to give them chocolate, tobacco, &c. One day I heard the boats being lowered very quickly, and on inquiring, I found that one of these poor fellows had escaped from his warder and was overboard. It was really marvellous with what rapidity the boat was got down, and he was brought back, struggling all the way. Here is a snapshot of the boat taken by one of the officers." The photograph showed a large expanse of sea, and a small boat going to the rescue. "Tommy overboard: Emergency Boat," was written underneath. "When we went down to see him next morning, he said, 'Oh, sister,' I did so enjoy my swim!'"

### SNAPSHOTS.

There were other photographs in the book; two were of the ship's staff, the officers in uniform, and the sisters in caps and aprons, though one is wearing a white sun hat, for the heat was intense. The queer little object on Miss Clapham's cap was, she explained, a chameleon belonging to one of the officers; it had just climbed on to her head when the group was taken.

"We hoped to go on shore at Las Palmas," she explained, "and we got up early on purpose, longing for a stretch after so many days at sea, but on account of plague in Cape Town we were not allowed to land."

Sisters Couper and Miller are described as "time-expired," but Sister Clapham expects to return in about sixteen or seventeen days to South Africa; though the Mines Hospital will not, of course, be opened until the country is more settled.

"I am hoping to see several of the London hospitals this time," she said. "I had an introduction to the London last time I was here, but there was not time for a visit. I want to do a good deal this time."

### Minor Appointments.

SOUTH WIMBLEDON AND MERTON COTTAGE HOSPITAL.—Miss Ada Emily Hocking has been appointed staff nurse. She was trained at the Tottenham Hospital, and has since been engaged in private nursing. She holds the L.O.S. certificate.

KINGSTON-ON-THAMES UNION INFIRMARY. — Miss Kate Parker has been appointed ward sister. She was trained for three years at the Birmingham Infirmary, and has been engaged in private nursing at Leicester. She holds the L.O.S. certificate.

BRADFORD EYE AND EAR HOSPITAL. — Miss Louisa Harrison has been appointed sister. She was trained at the Royal Infirmary, Sheffield, where she has also been sister for holiday duty. She has also been charge nurse at the Fountain Fever Hospital, Lower Tooting.

BURGH FEVER HOSPITAL, PAISLEY.—Miss W. Carpenter has been appointed sister. She was trained at St. George's Infirmary, Fulham Road, London, and the Fever Hospital, Southampton. She has since been assistant nurse at the City Hospital, South Grafton Street, Liverpool.

WESTON - SUPER - MARE GENERAL HOSPITAL. — Miss Kathleen King and Miss E. Roberts have been appointed staff nurses. Miss King was trained at the Cottage Hospital, Bromgrove, and has since been staff nurse at the Jubilee Hospital, Woodford Green, Essex. Miss Roberts was trained at the Meath Hospital and County Dublin Infirmary, and the Grimsby and District Hospital.

### Presentations.

STRATTON COTTAGE HOSPITAL.—Mrs. Norledge, late matron and nurse at Stratton Cottage Hospital, has been presented with a purse of gold and a box in recognition of her services.

### My First Week in a London Hospital.

By "An Old Pro."

It was on a Wednesday evening in the early autumn when I entered one of London's largest hospitals for a year's training, preparatory to beginning my career as a district nurse.

I had already received a certain amount of training in a small country infirmary, so that the feeling of awe, wonder, and even horror which is supposed to overcome the "new Pro" on her first introduction to hospital life was absent, and even the announcement of the bright young Sister who met me, and told meI was to begin my work in a men's surgical ward, did not quench my enthusiasm in the least. Nor did I wish to wait for the refreshing cup of tea she gave me in her own pretty room, before conducting me to my own and helping me to struggle into my new uniform.

### Introduction to the Staff.

I was then taken to the ward, through, as I thought, endless corridors and turnings, and introduced to the Sister and staff-nurse under whom I was to begin my work. Their greeting was kind, but decidedly cold. First impressions are not, however, everything, and I must say that no "pro" could have had a better or kinder Sister and nurse, firm and strict, but willing to teach and to help one over difficulties. After all, when one has had a very busy, long, tiring day, perhaps visitors, operations, an unexpected visit from the surgeon, or "taking-in," and, in a large hospital, new "pro's" arriving so frequently, can it be supposed we should come in for a very bright, hearty greeting? I say this to encourage some who may be tempted to feel—shall I say?—just a wee bit disappointed and discouraged at the outset of their hospital career by trifles. I found my fellow "pro" a cheerful, happy girl; so we made friends at once, and paired off to supper, a very pleasant, sociable meal, when all the day nurses were off duty till seven the next morning, and did not have to hurry back to the wards.

### The First Day.

I slept soundly that night, and woke in the morning quite fresh and ready for breakfast, when my fellow "pro" came at six-thirty to take me to breakfast. Seven o'clock, off to the wards, and then work—sober, practical work—in reality staring me in the face. Beds to make, floor to sweep, and dusting, washing, polishing for dear life till half-past eight, when we sat down for a quiet half-hour, to a kind of second breakfast or lunch. Next, milk had to be taken round to all the patients, their breakfast having been served at six, by the night nurse; dressings, &c., had to be put at the foot of each bed, on a small table, ready for the house-surgeon's visit. Those whose turn it was went off duty until twelve, the patients' dinner-hour. My fellow "pro" and I were off that morning, leaving the staff-nurse alone.

At twelve punctually we were back and dinner in full swing. The ward had to be again swept and dusted before we went to our dinner, after which we sewed, making bandages and padding splints, until three-thirty, when it was time to prepare the patients' tea. By the time that was done and cleared away, and we had partaken of our own, it was five o'clock, the hour at which the evening work began. Then we had beds to make, backs to wash and rub; the fomentations and dressings had to be changed, and the ward again swept and everything put straight. The lights were lowered at eight o'clock, and in this, as in all well-managed wards, the next hour till supper-time was occupied by us in reading, or working, or what we chose, as long as we were quite quiet and did not disturb our patients. That night, however, was the weekly service night, and oh! how much I enjoyed that little service you can scarcely imagine. It seemed so home-like and refreshing at the close of my first day in that large hospital, so far away from my country home. It was indeed a rest by the way, a happy ending to a happy day, and one that I shall never forget.

### On Duty Alone.

Friday and Saturday flew by, and when on Sunday morning Sister decided that I should remain on duty alone, while both nurse and the other "pro" went off, I was considerably elated; but, alas! pride was destined to have a fall that day. Nurse gave me many necessary instructions, and I was told to be quite sure to call the staff-nurse in the adjoining ward, to accompany and assist the house-surgeon and Sister on their round through my ward, and *not* to let the dinner things be late, but to have everything quite ready by twelve o'clock.

### How I was Hoaxed.

Well, all went on quietly till about a quarter to eleven, when one of the men begun, "I say, nurse, isn't it time you laid the cloth for dinner?" "Yes," chimed in another. "Sister will be cross if you are not ready when the bell rings, and the bread takes so long to cut." Very innocently and unconsciously, I took their hints and promptings, and by a quarter past eleven the table was laid for those who were up and the bed-tables each at the sides of those who were in bed, and I was congratulated on being ready in time, when a rustle at the other end of the ward attracted my attention, and in walked Sister and the surgeon, with the other nurse behind. Never shall I forget the visible amusement of the surgeon, and Sister's horrified looks, as she exclaimed, "What does *this* mean! Put them all away at once until the proper time." Accordingly, amidst the smiles of Sister and the surgeon, and the intense amusement of the patients under the sheets, who were well pleased at the unsuspecting way in which I had walked into their trap, I put them all back again. I did not so much mind that as I did the contempt with which I was regarded by that other nurse, and I am afraid I have not yet forgiven her.

### "Taking-in" Day.

Notwithstanding this slight mistake, my first Sunday was a very happy one—an hour off in the afternoon, and time to attend church in the evening. Monday morning came, busy and bustling as ever, rather more so than usual, as to-morrow was "taking-in" day, and there were extra dressings to cut, splints to overhaul and sort, fracture boards to be placed in readiness. When the next day arrived, and the first case was carried into the next ward, instead of ours, great was my indignation. But before the day was over I was anxiously asking when the "take-in" would end, as we were filling in so rapidly. I was not yet initiated into the ways and means of a large hospital, it seemed: some of our convalescents were to sleep in wards that had vacant beds, and had to be sorted out, as it were, some as out-patients, who were to come up certain days to see the surgeon and have their dressings changed, and the others to remain as regular inmates until the next take-in, a fortnight hence.

### Operating Day.

Wednesday was operating day, and I am afraid I did not appreciate my part in the work--that of sitting watching those patients brought back from the theatre until they fully regained consciousness and could be left with safety. I wanted to assist in the actual work going on around me, but one of the first, but by no means the easiest, lessons of hospital life to be learnt is obedience. Until we have well learnt that, all our other efforts will be in vain. So the week came to an end. Time has flown by, and yet, though it may sound contradictory, it seemed as though I had been there a month, so quickly can one get accustomed to a life given up to the service of others.

I should like to add how the influence of that dear Sister under whom I first worked, and grew to love, seemed to follow me as I moved from ward to ward; and when, at the close of my hospital life, I was back in the same ward for the last few weeks, words fail me to describe the joy it was to work again under her strict but kind rule. She is still busily engaged in the work she loves so well, but in South Africa instead of a London Hospital.

### District Nursing Superintendents in Conference.

BY A CORRESPONDENT.

A VERY pleasant day was spent at the Central Home, Liverpool, on February 19th, when twenty-one superintendents from the district nursing homes in the North of England met in conference. They came from towns as widely apart as Gateshead and Birmingham, and only three of those invited were unable to attend. The idea emanated from Liverpool, and this first meeting was in every way experimental. The hour for the preliminary proceedings was noon, and, to the great satisfaction and pleasure of all, Mr. and Mrs. William Rathbone were able to be present to welcome the visitors on behalf of the Liverpool Association.

Mr. Rathbone, in his earnest and inspiring address, emphasised the necessity for those engaged in the supervision of such an important work to keep ever before them and their nurses a high ideal, not merely the assuaging of physical suffering, but also the striving to carry on the work of our Lord and Master, who nineteen hundred years ago went about raising and cleansing both soul and body. He spoke of the work of the superintendent necessarily being isolated and therefore in danger of becoming narrowing. This intercourse and intercommunication would guard against waste of power by repeating experiments which have failed, for however clever a worker, even were she a Miss Nightingale, she could not evolve from her own inner consciousness all that the experience of others and the past could teach her.

After Mr. Rathbone's helpful and encouraging address some business was enacted as to the procedure for the afternoon, and a sub-committee was appointed to consider arrangements for future meetings. Miss Rosalind Paget had kindly come from London to give her counsel and advice, and she was unanimously and warmly asked to take the chair. Luncheon was served at 12.30, and after this there was time to see the new Central Home, the discussion of the subjects on the agenda being commenced punctually at 1.45.

Each subject was introduced by one of the superintendents, to whom a list had been previously sent. Seven were thus brought forward. One of the most interesting was the "best way of obtaining the right nurses for District Work." After much discussion as to the desirability of higher salaries being given and the greater inducements held out to private nurses, there was but one conclusion, that the great point was to get the hospitals to look favourably upon and understand the importance of district work as a powerful factor in benefiting the people, so that assistance might be obtained from them, and that some system might be organised whereby nurses before completing their fourth year of training might, if willing, be passed on to the district nursing associations to help in their work.

A bold member suggested that a memorial hospital should be built where all the nurses trained could be prepared for district work. The conference, with one exception, was unanimous in the opinion that nursing attendance at schools for the prevention of minor ailments was a work which should be undertaken by the associations, and carried on by nurses from the homes, in spite of the difficulties of arranging the nurses' work. In Liverpool, where this scheme has just been organised, a special "Schools Nurse" has been added to the staff of the central home, who in her spare time assists with the work of the districts whose nurses are off duty for week-ends, &c.

The discussion on the third point, namely, the reception of contributions, was long and animated, but again with one exception the meeting was unanimous that it was desirable that a nurse should both invite and receive contributions to the funds, only being punctilious as to an official receipt being sent by the superintendent for all donations.

The advisability of not changing a nurse's district was ably dealt with in a short paper introducing the subject; and after weighing both sides of the question and hearing various opinions, the members present decided that it was only advisable under exceptional circumstances to change a nurse from one district to another.

The arrangements of the various associations represented as to holidays, week-ends, and off-duty times showed some slight variations, but all felt that the work of the district nurse was one which made relaxation and off-duty times most important considerations.

As to the salaries of nurses, the superintendents present were strongly of opinion that no salary should be less than £30 to begin with, increasing to £40 or even higher if possible. The following resolution was unanimously passed:—"That committees be respectfully recommended, in view of the very small salaries nurses receive, to consider the question of assisting them in obtaining pensions."

In conclusion it was unanimously resolved that a yearly conference be held, and that the next place of meeting be Manchester.

A cordial vote of thanks was passed to Miss Paget, while the hope was expressed that she would be willing to preside on the next occasion when the matrons of the North meet in friendly conclave.

Tea at 4.30 brought the interesting day to a close, and as one after another left the Central Home, expressing their pleasure and appreciation of the opportunity afforded for mutual discussion and help, those who had inaugurated the conference, and especially the superintendent of the Central Home, who had made all arrangements for the entertainment of the visitors, felt more than satisfied that the new departure had been successful.

### Appointments.

BOROUGH OF REIGATE ISOLATION HOSPITAL.—Miss J. M. Dundas has been appointed deputy-matron temporarily during the indisposition of Miss Amcoats, the matron.

SUNDERLAND AND DURHAM COUNTY EYE INFIRMARY.—Miss Elizabeth Cuthbertson has been appointed matron. She was trained at Monkwearmouth and Southwick Hospital, where she has since been charge nurse.

EXETER UNION HOSPITAL.—Miss Georgina Bouch has been appointed superintendent nurse. She was trained at Manchester Royal Infirmary, and has since been superintendent day nurse at the Camberwell Workhouse, East Dulwich, S.E.

ST. GEORGE'S HOSPITAL, BEYROUT, SYRIA.—Miss E. R. Wortabet has been appointed lady superintendent. She was trained at the Middlesex and London Temperance Hospital, and has had experience in workhouse and district nursing. She holds the L.O.S. certificate.

SPITTLESEA INFECTIOUS HOSPITAL, LUTON.—Miss Annie Alexander has been appointed matron. She was trained at Salisbury General Infirmary and the London Fever Hospital. Her previous appointments have been matron at Colchester Infectious Hospital, matron at Brentwood District Cottage Hospital, and matron at Brighton College. She has also been engaged in private nursing.

BIRMINGHAM AND MIDLAND HOSPITAL FOR SKIN DISEASES.—Miss A. M. Browne has been appointed matron. She was trained at the Royal Infirmary, Edinburgh. She has since been engaged in private nursing, and has also been sister at the London Hospital and the Seamen's Hospital, Greenwich, lady superintendent and secretary of the Mother's Seaside Convalescent Home at Herne Bay.

### Echoes from the Outside World.

AN OPEN LETTER TO A HOSPITAL NURSE.

THE penalties of Royalty must at all times be more or less trying, but they seem to me to have been especially emphasised lately in the matter of the King's visit to the Empress Frederick. Because a brother wishes to see his invalid sister, whose health is admitted to be in a precarious condition, and to convey to her personally the last words and actions of that mother whom they loved so well, a certain set of ill-mannered journalists endeavour to convince their readers that the journey, though ostensibly fraternal in its character, is in reality political, and is simply an excuse for discussions and plots, machinations and alliances between the two sovereigns of two great European Kingdoms. I am glad to say that the German, not the English, newspapers are the chief offenders, and that a great number of the King's subjects assembled around Charing Cross Station on Saturday evening, although, of course, no State was observed, to show the sympathy of the English nation. The King took his favourite dark-brindled bulldog "Peter" with him as companion; otherwise his suite consisted simply of two equerries and his medical adviser. He was met at Frankfurt by his nephew, the German Emperor, at a quarter to nine, both monarchs having already breakfasted. Together they continued the journey to Cronberg and drove to the Schloss Friedrichshof in a sleigh drawn by a pair of greys. The whole of the country around the castle is covered with snow, and sleighing is universal. Having escorted the King to the door, the Emperor returned to Homburg, from whence he visits his mother every day. The last reports speak more favourably of the Empress Frederick's general condition, though if, as it is stated, she is suffering from cancer of the kidneys alleviation of pain is necessarily the most that can be hoped for. At present the attacks of pain are very severe, rendering the patient indifferent at the time to all going on around her, and during one of these paroxysms she was heard to murmur, "Oh, how he must have suffered!" evidently referring to the last illness of her husband. But when the fiery trial has passed for the nonce, she goes out in the grounds, reads and paints, and is even pushed in her bath-chair through the apartments on the second floor which are reserved for her guests. Princess Beatrice is also about to pay her sister a visit on her way to stay with the Empress Eugénie. When the King returns he will probably remain some little time in London, as there must be so much business he has to transact. It is not at all surprising that he has been obliged to inform the Lord Lieutenant of Ireland that he must put off his Irish visit till next year.

IT is quite possible that by the time this reaches you the result of much labour and, alas! much loss of life may have resulted in definite news of a cheering character from South Africa. There is no doubt that De Wet is hard pressed. Although his name would suggest otherwise, he hardly seems happy between the two flooded rivers, and his having to fly before Colonel Owen, leaving two of his guns in our hands, must have had a dispiriting effect upon his men. Still, of course defeat is not capture, and till the poor fellow—for whom one cannot help feeling sorry, knowing he has lost everything in the war, wife, children, home, and independence —is in our hands the constant skirmishes will continue. Botha, too, has no success to cheer him, and the "attempt on the part of the Boers to invade Cape Colony has evidently failed." Even the wisely reticent Lord Kitchener makes this admission.

IT will probably interest you to know that a new league has been formed. It is yet in its infancy, as its name, "The Twentieth Century League," denotes, and its object is almost as novel as its name. The League has been started under the auspices of the Bishop of Stepney, the Bishop of Kensington, Sir Henry Burdett, Mr. W. B. Dickinson (the Chairman of the London County Council), the Lord Mayor, and others. Earl Roberts, though unable through press of official work to be present at the Mansion House on the day of the meeting, wrote to say how much he approved of, and sympathised with, the objects of the gathering. The enthusiasm likely to be thrown into the work of the League should be materially increased when those who are tempted to treat it with indifference reflect that it was one of the great desires of the late Bishop Creighton to ameliorate the social condition of the poorer people of the metropolis, and many who help might look upon any assistance, either personal or pecuniary, which they are able to render as a tribute to the memory of one whom we all loved and revered. The *raison d'être* of the Twentieth Century League is to endeavour to find some wholesome outlet for the exuberance of animal spirits which has led to that state of disorder in some parts of London which has been termed "Hooliganism." I write the word diffidently because Sir Henry Burdett made a point of asking the Press not to refer to street ruffianism by a name which has about it such a grand sound that it is in its very self attractive to a certain set; but I must mention it just this once so that you may understand the class of boys and girls whom it is proposed to benefit. Those who have already become street ruffians in the worst sense must be left to the police, but it is maintained that it should be possible to get hold of boys and girls on leaving school, and by providing them with means of adequate exercise and rational amusement prevent many from drifting into bad habits. This could be best done by means of gymnasiums, lads' brigades, homes, and clubs, male and female.

ONE point will, I think, appeal very strongly to all commonsense men and women. The £5,000 asked for is not to be expended in the formation of new institutions: it is to be applied to the support of existing organisations, that they may multiply their opportunities for good and attract many who at present are allowed to slip away for want of money and workers. Later on, if the League prospers, it may widen out so as to include new fields; but it is pleasant for the time to denote its cosmopolitan character, and that upon its working committee there are members of the Young Men's Christian Association, of the Oxford House, of the Federation of Working Boys' Club, of Toynbee Hall, of the Church Army, the Jewish Lads' Brigade, and the Social Institutes Union. A member of the School Board said that the most powerful means of arresting the spread of street ruffianism was the evening continuation schools. I fear that in their present condition the increase of learning is not the desire of these unruly spirits; the object of their lives is "fun," as they call it, and nothing but sports and games will in the first instance be attractive enough to compete with the joy of street fights. Later on, maybe, as the French say, "the appetite will come with eating," and those who were once unfit for wholesome food will be glad of solid fare.

TWO correspondents have written to draw my attention to the fact that Lady Beatrice Butler was spoken of last week as a "descendant" of Queen Elizabeth, whereas that Sovereign was never married! I humbly apologise to the shade of the injured Queen, and beg to state in a most contrite frame of mind that I never meant to malign her. I only meant that Anne Boleyn and Lady Beatrice were descended from th same ancestors.

### City Orthopædic Hospital.

LECTURES TO NURSES.

Mr. Noble Smith, Senior Surgeon to the City Orthopædic Hospital, in his second lecture of the session to nurses, first referred to his previous lecture in which he had urged the importance of recognising the difference between inflammatory diseases of the spine and joints (generally tubercular) and non-inflammatory affections. Continuing this subject, Mr. Noble Smith remarked how difficult it was for the surgeon, even one who had long studied these diseases, to form a definite opinion during the early stages of an affected spine. In incipient caries there might be a slight rise in temperature, 1° to 2°, or this sign might be absent; it could never be relied on. Pain, so characteristic in the majority of cases, might be entirely absent. He had known caries to proceed so far without pain that nothing wrong was recognised until considerable deformity had occurred or an abscess had formed.

The lecturer cautioned nurses to watch all cases of weak spine very carefully, and to inform the doctor of everything they noticed. The diagnosis of a disease was undoubtedly the surgeon's business, but there was no reason why the nurse should not help in this matter. The nurse was with the patient when the doctor was away, and she ought to use her intelligence in observing symptoms. For that reason she should know something about diseases, so as to realise what to look for.

Because of the difficulty of diagnosing caries from simple weakness, and because caries is almost always accompanied in the early stages by weakness, and often by some lateral curvature, therefore it is injudicious to commence any exercise treatment in the case of a weak back, until the surgeon could be certain as to the nature of the case. He had seen caries most rapidly developed by exercises given to a child who seemed to be suffering from lateral curvature or weak back alone.

Therefore the lecturer advocated great caution. Very weak children could not bear much exercise with benefit, and it was better at first to use a light support, as they did at that hospital. Subsequently, when the patients had improved, a result which almost invariably happened, then some carefully regulated exercises might be carried out with benefit.

These exercises required skill and care in devising and in carrying them out. No two patients were exactly alike, and the surgeon must himself determine what exercises should be done.

Then as to the question of pressure sores. These might occur as bed sores, or from undue pressure of a splint or apparatus. Much depended upon the nurse in preventing the occurrence of bed sores, and generally they could be avoided. Strict cleanliness, shifting of the patient's position, keeping the skin dry, relieving pressure at any one place by supporting the parts around with pillows or cotton-wool, and the use of various spirituous or greasy applications. These means were, or should be, known to every nurse, but it occasionally happened that a bed sore would occur. In paraplegia, where the nerve supply of the legs is cut off, bed sores are very difficult to prevent. Even in a case of osteotomy of the femur, which was now in the wards—the patient being very thin, and his skin very tender—a sore had formed in spite of every care to prevent it, but that was not likely to cause much more trouble, as the bone was uniting firmly in its improved position, and in a few days the enforced rest on the back would be relieved.

In applying splints in the treatment of club foot the production of sores should be avoided, but it sometimes happened that after tenotomy in a case of severe deformity, considerable pressure had to be applied in order to secure the best position. Under these circumstances, the nurse ought to watch the case very carefully. If the child complained of pain for half an hour or more continuously, then the splint must be loosened or reapplied. It sometimes happened that after such complaint on the part of the patient the pain might cease, and the nurse was apt to think that the trouble was over, but such was not the case; the cessation of pain was caused by a numbing of the cutaneous nerves from the continued pressure, and when the splint was removed, perhaps the next day, the skin might be found ulcerated at the point of pressure.

After tenotomy there should be no pain or only a trivial amount, passing off in about half an hour. If it continued longer, then the splint should be loosened or readjusted.

Mr. Noble Smith's next lecture will be given at the Hospital on Wednesday, March 5th, at 3.30 P.M. The subject is "The Nursing of Orthopædic Cases," and the lecture will be illustrated by casts from the Museum.

### Everybody's Opinion.

**** The author of "Nursing Up to Date" is requested to send name and address.*

### A WARNING.

"Matron" writes: The facts related headed "Warning," in last week's Hospital, are simply identical with what took place at Romford Cottage Hospital, Essex, on the evening of November 6th, 1900 The patient was admitted and detained a few days. Some pity was felt for him as attempted poisoning was suspected owing to his being out of employment, the means causing such pain that he became frightened and so applied for relief."

### SO-CALLED TRAINED NURSES.

"A Trained Nurse" writes: I am very glad "M. M. M." is doing her best to expose the fraud now practised so much—not only by males, but by females also. I should like to see the subject fully discussed in some daily papers. One thing however we can do, namely, take our testimonials to every case and show them. The public would then expect this as a right in a short time, and no good nurse would mind doing it. I have done so for years, and I have benefited by it in every way.

### HEATING APPARATUS WANTED.

"Perplexed" writes: Would some reader of your columns suggest any kind of heating apparatus for a steriliser wanted for district surgery. The steriliser would hold about three quarts of water, and therefore would require a fair heat power to keep boiling. In many houses in Maryport the people do not burn gas, hence the objection to an otherwise simple solution of the difficulty; and I want the thing to be a perfectly useful article under any circumstances. To keep a large spirit lamp in frequent use might prove somewhat expensive, and an oil stove would smell.

### NURSES IN INDIA.

"Placidity" writes: Having had many years' experience in India, I would add my warning to nurses intending to try their luck there. There is plenty of work, and moderate pay; many kind friends as elsewhere, but no pagoda tree to be shaken. The "pucka" Englishwoman is expected to set an example of hardihood and strength to her dusky sisters, and women who go out to India to earn a livelihood as nurses are despised—and rightly so—if they assume the pose of a moneyed aristocrat. There are thousands of poor whites in Indian cities with no higher pay than 150 rs. per mensem—which is the highest rate a nurse working on her own account can hope for—who cannot afford, and therefore do not get, carriages, punkahs, &c. I have known an English nurse receiving 150 rs. and living on it in a comfortable, happy manner, walking to her hospital daily twice, the distance being nearly two miles, having to do night duty as well as day occasionally, and yet treated as a social equal by the very highest officials, and eventually becoming the wife of one of them. Peevishness and discontent will not help us. Send the right sort to India, and they will make their way without injuring others or themselves.

### OVERWORKED ARMY SISTERS IN SOUTH AFRICA.

"COLONEL B. SOMERVILLE-LARGE, of the Army Medical Corps, P.M.O., of No. 6 General Hospital, Johannesburg," writes: A letter in your much-valued weekly dated December 29th, 1900, has been brought to my notice by several nursing sisters, No. 6 General Hospital—ladies who have been doing duty in my hospital since its arrival in South Africa—who are indignant at the inference to be drawn from it, namely, that the nursing sisters are being broken down from over strain and hard work. On their behalf as well as on account of the reputation the hospital has gained in this campaign, I write to give the statement made in that letter a "complete denial." Every one of the sisters that have left my hospital have expressed both to the Superintendent Sister and myself their high appreciation of the way they had been treated, the pleasant time they had while doing duty at the hospital, and how sorry they were to leave. True, Miss Asman was invalided home from ill-health due to a very delicate constitution. She as well as the three other Princess of Wales's Sisters whose names are mentioned in the letter, and who through necessity of holding appointments in England had to leave before the war was over, all expressed themselves as above, and were in good health when they left, and I am thankful to say that Miss Peers and Miss Burdett are in the best of health and performing their work to the best of their ability. Reports of this sort, unless publicly denied, tend to cause discontent throughout the "Army Nursing Reserve Service," also to reflect adversely on the administration of the authorities, who have performed such duties splendidly through a long trying campaign. Consequently I write trusting you will insert this letter in your next issue. It might be as well, in order to allay the minds of the friends of these ladies at home who may have read your letter, to mention that they reside in large comfortable houses, furnished with every luxury; their dietary is carefully looked after, and they suffer no hardships. You have perfect liberty to publish my name as I consider unsigned documents unsatisfactory in print.

### THE MYSTERY AT NEWTON ABBOT.

"MISS CECILIA FLAHERTY" writes from the Infirmary, Newton Abbot: I have read your remarks on my case with much interest, and I wish to thank you for the fairness and justice you have shown. I should like to give you an outline of the duties I was expected to perform when the patient complained of was at her worst. There were six cases of ringworm (children) which required dressing at 8.30 P.M. Several 4-hourly dressings, medicines, and occasional poultices, in the male ward, which also contained two mental cases. In the female ward there were six mental cases, most of them excessively troublesome at night. One was subject to fits and was practically dying at the time. Then in a side ward I had two bad cancer patients, one of them also an imbecile. The other patients were either ill or infirm, and there were ten or twelve children. For assistance I had an old woman over seventy years of age, who generally sat in the large ward where the most troublesome cases were. The patient in question had been very trying for about two months; and as she was becoming worse the doctor spoke of moving her to a lunatic ward at the house, previous to sending her to the asylum. However, she was put into a side ward where three other patients were (one of whom complained that her baby was almost thrown into convulsions by this old woman's noise). When I went on duty at 8 P.M. she was simply raving mad. I suppose she was unsettled at being moved, and she was constantly getting out of bed and stripping the bed of the patient next her, shouting the whole time. I asked the superintendent for extra help, saying "I could not take the responsibility of this case, and do my other work as well." So the wardswoman was taken from the large ward to sit with this patient, while I divided my time among the others. When the doctor came he also refused to grant extra help, on the ground that "he did not consider it necessary," and said that I could send for him again if patient became more violent. Therefore, as I was refused assistance when I applied for it, and wanted it most, nothing remained but for me to do the best I could under the circumstances. I did not receive definite orders from the doctor that patient was not to be left at all; had I done so the order would have been carried out unquestionably, although the other patients would have been neglected in consequence, and I should still have been in the wrong. I was supposed to have received this order on January 12th, and I knew nothing of it until I saw the Visiting Committee on January 16th, when I was more than astonished to find myself charged with refusing to carry out the doctor's orders. On January 13th arrangements were made for me to have other assistance with the morning's work so that my wardswoman could remain with patient; and after that, an attendant was sent up specially to sit with her. The most mysterious and damaging reports were circulated about me, and I wrote to the Board asking for an investigation. As I have been here nearly two years, and am well known, I preferred that the true facts of the case should come before the public while I am here to defend my good name.

### "TWO CONCEPTIONS OF A NURSING VOCATION."

"A MATRON OF EXPERIENCE" writes: Will you allow me space in your valuable paper to say a few words with regard to your note headed "Two Conceptions of the Nursing Avocation," the logic of which is somewhat odd. The rector of Christ Church, Kingstown, sets forth a very super-natural, or shall we say un-natural, standard for the very prosaic though honourable avocation of nursing. May I ask whether the clergy themselves take as exalted a view of their own profession, which is admittedly a far more strictly religious one than either the medical or nursing professions? I trow not! But, given that they do, does the argument hold good that because doctors or clergymen have a high conception of their offices they are therefore to be content to be relegated to the housekeeper's room to have their meals with the upper servants instead of being asked out to dinner and received in society as gentlemen? I do not think from my personal acquaintance with the clergy (which is somewhat large) that a clergyman would call it "the temper which is absolutely inconsistent with his avocation" were he to object to dine with his uncle's butler instead of at his uncle's table, however well educated the butler might be! Many nurses are in the habit of dining late as a matter of course, and when "off duty" of dining out, possibly even with the aristocracy, and to ask a nurse of this class to have her meals either in the kitchen or housekeeper's room is a gross insult. It is quite easy to arrange for a nurse to have her meals in the dining-room, either before or after the family have had theirs—as I know by experience in my own home—and I have more than once seen this arrangement made for nurses in hotels. If the nurse be a lady she would very much prefer not to have her meals with the family where she is nursing, or at table d'hôte in her nursing uniform, but it is decidedly nasty for a healthy person to be obliged to eat meals in a bedroom. "Straws show which way the wind blows," and the incident under discussion has a significance of a more serious nature than appears on the surface. There is a tendency, unfortunately, in the present day among men of the world, and—I am ashamed to say it—the clergy too, to rank a nurse either as a nun or a demi-mondaine the former is supposed to require none of those social amenities which make existence tolerable, the latter neither respect nor consideration. Both these conceptions of the true nurse are, of course, utterly and equally erroneous. To be a good nurse of necessity predicates being a true woman. A nun is, of choice, an unsexed and unsympathetic nonentity, and therefore could never make a sensible, sensitive, or sympathetic nurse. But if a nurse is not a nun she will nevertheless certainly not allow herself to be treated in a light or disrespectful manner, nor ought she to be so indifferent to her own womanly dignity as to be willing to be treated with the insult and contempt implied by the man (a gentleman he cannot be called whatever his social position) who told the nurse she was to have her dinner with the housekeeper. It is not to be wondered at that she left him, for a man who could treat her with such indignity would not have been a safe patient for any honest woman to nurse.

### For Reading to the Sick.

LOVE DIVINE.

O LORD, how happy is the time
When in Thy love I rest!
When from my weariness I climb
E'en to Thy tender breast!
The night of sorrow endeth there—
Thou art brighter than the sun;
And in Thy pardon and Thy care
The Heaven of Heavens is won.
—*George Macdonald* (Translation).

If one heart in perfect Sympathy
Beat with another, answering love for love,—
Weak mortals, all entranc'd, on earth would lie,
Nor listen for those purer strains above . . .
THOU know'st our bitterness!—our joys are Thine!
No stranger THOU to all our wand'rings wild!
Nor could we bear to think how every line
Of us,—Thy darken'd likeness and defil'd,—
Stands in full sunshine of Thy piercing eye,
But that THOU call'st us Brethren! Sweet repose
Is in that word;—the LORD who dwells on high
Knows all, yet loves us better than HE knows.
*Keble.*

God would lead us into a life of union with Himself; but first He must loosen the ties which bind us to earth, and so in some way or other sorrow comes to us. It may be that He lays on us His own afflicting hand, and by sickness cuts us off from all that up to this time had given us joy; or it may be that He sets us face to face with our sin; but one way or another God is ever . . . making us poor that He may make us rich.—*G. Body.*

You can be "acquainted with God," you can read in creation, you can read in the incarnate Christ, His character and His power; and thus it is that the illuminated soul of the creature has some faint but true glimpse of the "glory of God." . . . For to *see* what is beautiful is, by the privilege of that great nature within us, to *love* it; and thus to be "acquainted with God" is to proceed to love God, and what is this but more and more to enter into the mystery of His glory?—*W. J. Knox Little.*

Blessed is he that loveth Thee, and his friend in Thee and his enemy for Thee. For he alone loses no dear one to whom all are dear in Him who is never lost. And who is this but our God, the "God that made heaven and earth" and filleth them, because by filling them He created them? Thee none loseth, save he that forsaketh. And whoso forsaketh Thee, whither goeth he but from Thee smiling to Thee frowning? —*S. Augustine.*

Ah, dearest Lord! to feel that Thou art near
Brings deepest peace, and hushes every fear;
To see Thy smile, to hear Thy gracious voice,
Makes soul and body inwardly rejoice
With praise and thanks.

We cannot see as yet Thy glorious face,
Nor yet our eyes behold its love and grace,
But Thee our inmost soul can surely feel,
Oh, clearly, Lord, canst Thou Thyself reveal,
Though all unseen.
*Christian Gregor.* 1778.

### Notes and Queries.

**The Editor is always willing to answer in this column, without any fee, all reasonable questions, as soon as possible.**
**But the following rules must be carefully observed:—**
**1. Every communication must be accompanied by the name and address of the writer.**
**2. The question must always bear upon nursing, directly or indirectly.**
**If an answer is required by letter a fee of half-a-crown must be enclosed with the note containing the inquiry.**

*Home.*

(237) Is there any home which would receive a lady, aged 46, and a gentleman, aged 40, at very low terms? They are the children of a clergyman, and mentally feeble though not requiring special care. Their only means of support is a charitable grant of £25 a year to the lady, and one of £20 a year to the man.—*Mrs. G. P.*

You might write to the National Association for the Welfare of the Feeble-minded, 53 Victoria Street, S.W. Ladies are taken from 10s. 6d. a week at St. Mary's Home, Painswick, Glos.

*Army Nursing Reserve.*

(238) Kindly tell me how to enter my name on the Army Nursing Reserve.—*Nurse.*

Write for application form to the Hon. Secretary, 18 Victoria Street Westminster, S.W.

*Beef Tea.*

(239) What is the truth about beef tea? Is properly made beef tea more than a stimulant, and what is "properly made"? Are Bovril, Liebig, &c., nutritious?—*Hey. What?*

Two articles on this subject giving full information appeared in our columns on December 29th, 1900, and January 19th, 1901.

*Nursing Home.*

(240) I am seeking a partner with a little capital in order to develop my house as a nursing home. I have many advantages, but I am not a trained nurse. Can you kindly advise me as to a good agency, or as to the best means of obtaining what I want?—*Nurse Alice.*

You could not do better than advertise in THE MIRROR and read our Query column regularly. Note our constant caution to nurses on the dangers connected with this form of enterprise.

*Army Nursing Reserve.*

(241) Will you tell me if any more nurses are required by the Army Nursing Reserve for South Africa? 2. And also give me the address of the Hollond Institute?—*A. S.*

Apply to the Hon. Secretary, 18 Victoria Street, S.W., for information regarding the A.N.R.: and to the Nice Nursing Institute, Villa Soleil, Avenue Thiers, Nice, for particulars of the late Hollond Institute.

*Asylum Training.*

(242) Will you kindly tell me how I can get into a hospital or asylum, where I should be well trained as a nurse, without paying premium, and receiving a small salary?—*E. P.*

Buy a copy of the "Nursing Profession: How and Where to Train," and it will tell you all about it. We should advise you to take your three years' certificate from a general hospital or infirmary before entering upon mental work.

*Training.*

(243) Is the Sheffield General Infirmary a recognised training school for nurses? I understand the term of training is only for two years.—*F. A. C.*

There is no institution styled the Sheffield General Infirmary. The Sheffield Workhouse Infirmary gives a three years' certificate. The Sheffield Royal Hospital and the Sheffield Royal Infirmary, however, only give one for two years. It is always wise of probationers to train for a three years' certificate.

*Army Nursing.*

(244) I should feel much obliged if you would tell me where I can get particulars for hospital nurses desirous of joining the Army Service.—*F. L.*

Address the Under-Secretary of State, War Office, Pall Mall, S.W., for particulars of admission into the Army Nursing Service.

*Military Convalescent Homes.*

(245) Can you give me particulars of the hospital which is being built, in Hampshire I believe, for the reception of some of the wounded from South Africa? To whom should I apply for a post there?—*Lucy.*

The nursing staff will probably be drawn from the Army Nursing Service and the Nursing Reserve. See reply to 238.

*Disinfected.*

(246) Would it be right of a nurse who has been attending a scarlet fever case to return to a house where there are other nurses without going into quarantine? Is the disinfection she takes in the house of the patient sufficient?—*Nurse S.*

It would be right if she took care that the disinfecting process in the patient's house was effectual.

**Standard Books of Reference.**

**"The Nursing Profession: How and Where to Train." 2s. net; post free 2s. 4d.**
**"The Nurses' Dictionary of Medical Terms." 2s.**
**"Burdett's Series of Nursing Text-Books." 1s. each.**
**"A Handbook for Nurses." (Illustrated.) 5s.**
**"Nursing: Its Theory and Practice." New Edition. 3s. 6d.**
**"Helps in Sickness and to Health." Fifteenth Thousand.**
**"The Physiological Feeding of Infants." 1s.**
**"The Physiological Nursery Chart." 1s.; post free, 1s. 3d.**
**"Hospital Expenditure: The Commissariat." 2s. 6d.**
**All these are published by THE SCIENTIFIC PRESS, Ltd., and may be obtained through any bookseller or direct from the publishers, 28 and 29 Southampton Street, London, W.C.**

### Travel Notes.

BY OUR TRAVELLING CORRESPONDENT.

### LXVIII.—HOLLAND (*Concluded*).

### THE THIRD DAY.

LEYDEN will take you, more or less, a whole day, leaving you the evening, perhaps, to visit Dordrecht, or some other place near by.

Leyden is about twenty miles from Rotterdam, and there are plenty of trains during the day.

There is much here for the artist and for the all-devouring kodak. The Burg is a very singular structure (admission 10c.) It is mentioned in history as early as the tenth century, but is undoubtedly of much earlier origin. I am sure you will go to the church of St. Pancras, if only to look with admiration at the last resting-place of gallant Pieter van der Werf, the undaunted defender of Leyden from 1573 to 1574; he died in an honoured old age in 1604.

The Stadhuis is full of interest, both inside and out; it was much altered and almost entirely rebuilt after the siege of 1573, but I love to go there and read the inscription over the side entrance, recording that terrible year of famine, and to think how often chivalrous Van der Werf must have gazed on those rising walls after the stormy year of the past.

### THE MUSEUM—WEIGHHOUSE AND BUTTER-MARKET, &C.

Leave the Museum alone and pass on to the picturesque Weighhouse and Butter-market in the Aalmarkt, untouched, I believe, since 1658. It makes a charmingly artistic subject either for the pencil or kodak. The University of Leyden is of considerable importance. The Prince of Orange offered the citizens exemption from certain taxes as a recognition of their splendid bravery and endurance through the siege, but they begged the gift of an University instead, which has been the educational cradle of many distinguished scholars. The church of St. Peter is the resting-place of the Reverend John Robinson, who was the leader of the little band of Puritans who left England to escape persecution early in the seventeenth century, and who eventually went to America in the *Mayflower*. John Robinson did not join them, but died in Leyden in 1625. Over his grave is a representation of the *Mayflower*.

If you can spare time, it is worth while to take the steam tram to Katwyk Aam Zee, six miles distant, because there you see the wonderful system of dykes and sluices by which the safety of Holland is insured from the ravages of the sea. The journey occupies forty minutes, and if you can spare the necessary two hours, you will be interested in seeing these wonderful engineering works.

### THE FOURTH DAY

may be spent at Utrecht entirely if you are much interested in ecclesiastical architecture. The cathedral is grand, though in a sadly mutilated state from the loss of the nave, which fell in (1674) during one of those violent hurricanes which sweep so destructively over the flat unprotected Netherlands.

Utrecht is a very ancient city. We find the first Christian Church established there in the seventh century. Chantrey Churchill, in his interesting little guide book, tells a delightful story of the attempted conversion of Radbod, the Frisian chief, by the proselytising Bishop.

Their chief Radbod was about to be baptised, and had in fact already placed one leg in the water, when the thought of his ancestors flashed across his mind. "Where are my dead forefathers now?" he asked of the Bishop. "In Hell, with all other unbelievers," was the uncompromising answer. "Then will I rather feast with my ancestors in the halls of Woden than dwell with your little starveling band of Christians in Heaven!" exclaimed the Pagan chief, and promptly withdrew his royal leg.

The cathedral is dedicated to St. Martin, and was founded on the site of the original church built by the uncourtier-like Bishop, the would-be baptiser of Radbod. There are splendid cloisters which connect the Cathedral with the University. The bells which chime in the lofty tower are forty-two in number; the principal one, cast in the fifteenth century, weighs 8½ tons. There are several other churches of interest, and the Paushuizen, now used as a telegraph office should be visited.

### THE FIFTH DAY.

Baedeker says two hours is long enough to devote to Gouda. Tastes differ; I spent three days there! I do not think there is any church on the Continent that has such magnificent stained-glass windows. The glass in our own Winchester College somewhat reminds me of it; but there the amount is so small. I think there must be at least sixty windows in the Church of St. John at Gouda. Two brothers named Crabeth executed the finest in the years 1555-56-57. The only object of great interest after the church is the Stadhuis (1449) and one may add the Weighhouse and Meat Market.

### THE SIXTH DAY.

This must be devoted to Delft, connected in the public mind with fine old pottery. It is very difficult now to pick up genuine specimens even in Holland. There is still plenty of mediæval architecture to be seen here, though the nineteenth century has robbed it of much of its interest. The first place to visit will be the Prinsenhof, the scene of the murder of William the Silent. This building, once a monastery, had been turned into a palace for the Prince of Orange, where he was living at the time of his death with his wife, Louise de Coligny. Alexander Farnese had offered a reward of 25,000 golden crowns to anyone who should rid the Spanish King of his formidable antagonist. Balthasar Gerhard, either actuated by love of money, or, let us hope, by a bigoted belief in spiritual benefit to himself, by thus ridding the world of the Protestant hero, shot him dead as he was descending a staircase into the gymnasium court, as it is now marked. It is always said by contemporary historians that Louise de Coligny had noticed Gerhard, felt an instant intuition that he meant evil to her husband, and begged him to beware of one whose over-zealous Protestantism excited her suspicions. Balthasar was executed with all the demoniacal horrors attendant upon justice in that age, and his family claimed the 25,000 crowns. The murdered prince was buried in the Nieuwe Kerk, where a magnificent monument guides you to his last resting-place.

You will like to see the Oude Kerk, which contains the monument of Admiral Maarten Tromp, who died off Scheveningen in 1650. It is not quite pleasant to remember that it was Tromp, who, after defeating Admiral Blake, fixed a broom to his masthead as a token that he had swept the seas of us. However, it is an English characteristic to be able to admire a gallant enemy, and most assuredly Tromp was that.

I must now bring these notes on South Holland to an end, hoping some day to travel with you in spirit over the more northern part. I have left you a clear day of your week to fill up in revisiting any place that has interested you, or in seeing something fresh.

### TRAVEL NOTES AND QUERIES.

KNOKKE, IN BELGIUM (Londoner).—There is another hotel, indeed three or four, nearer the sea; but they are all considerably more expensive. Their names are—Hotel de Knokke, Hotel du Phase, Hotel des Familles, and Hotel des Dunes. The roads are on the whole good for cycling, though not comparable with those of France. Full information on Knokke was given in the NURSING MIRROR for September 1st, 1900. Send 3d. to the Manager and it will be sent to you.

PARAMÉ FOR WINTER (Fighting-Fifth).—No, I could not recommend it; bleak to a degree in cold weather, and unpicturesque *à faire peur*. For winter, if you wish to be in that region, why not try Dinan, 18 miles inland, mild in climate, cheap living, and even a little English society if you care for it. Go to the Hôtel de la Poste for a few days, where the terms are very reasonable, and look for lodgings. I think you will find it altogether preferable to the sands at Paramé.

ROME OR DRESDEN FOR THE WINTER (Minna).—These towns are so widely different that it is difficult to advise you. If, as you seem to suggest, musical education is your object, then by all means choose Dresden; there is a larger choice of apartments than in Rome, and, taken all round, they are cheaper. Putting aside the question of music, Rome is preferable as a winter city; there is much more of general interest in it, and it is warmer and more sunny. Dresden is very cold in winter.

TOUR IN BRITTANY (Robertson).—Write me again a few more particulars. Especially how much each nurse can spend and the exact limit of their holiday; the time at their disposal makes such an important difference in arrangements. Also tell me whether they prefer quaint old towns or landscape scenery, and whether the sea coast or inland is most to their taste. Another thing I should like to know is whether they wish to keep moving continually or are content to remain, say, in only two towns and making excursions thence. Constant change of hotels greatly increases expense. If you will write me again immediately you see this I can answer in next week's paper.

